# How Do Perceived Health Threats Affect the Junk Food Eating Behavior and Consequent Obesity? Moderating Role of Product Knowledge Hiding

**DOI:** 10.3389/fpsyg.2022.836393

**Published:** 2022-02-21

**Authors:** Yanxia Li, Xiaohong Li, Tuanting Zhang, Haixia Guo, Caili Sun

**Affiliations:** School of Physical Education, Xian University of Architecture and Technology, Xi’an, China

**Keywords:** obesity, junk food, eating patterns, knowledge hiding, perceived threats

## Abstract

The predominant use of junk food in our societies is continuously held responsible for the obese body physiques and overweight among the kids and adolescents. The current supportive environments where organic foods are limited, and new processed foods have been brought to the market with more variant tastes and acceptability for the kids and adolescents that have diverged their eating patterns. It has significantly contributed to the health issues and growth discrepancies of the users. However, the awareness of the food contents is an important milestone for understanding the risks associated with the usage of junk foods. A quantitative approach has been used in this study to measure the effect of perceived severity, vulnerability and fear on the junk food eating behaviors and ultimately on the obesity. The moderating role of product knowledge hiding has also been measured on the relationship of junk food eating and obesity. Structural equation modeling is used using the software Smart-PLS for measuring the hypothesis with a sample size of 228 selected through purposive sampling. The sample consisted of kids and adolescents who were reached on purpose for data collection. The current study has explored the role of perceived severity, vulnerability and the fear of using junk foods which have been found as a negative effect on junk food eating behavior which is positively associated with obesity among the kids and adolescents. The result of study shows that perceived threat has a negative effect on the junk food eating behavior of the adolescents. However, the positive relationship of junk food eating behavior with obesity can be decreased if the information about the products is not hidden. This study will be useful for making the consumers aware of the product knowledge hiding of the junk food usage. Moreover, it will help the users in creating understanding of risks allied with the use of junk food which may be addressed in order to avoid obesity issues in the kids and adolescents globally.

## Introduction

Although there is a growing public interest in health, the rising incidence of diet-related non-communicable disorders, such as obesity, can still be seen. Obesity treatment interventions have had relatively limited impacts over time, especially in high-risk groups (Stice et al., 2006). While interventions to minimize sedentary habits, notably television consumption or spending time watching television, have been more successful, studies show that a greater knowledge of healthy eating determinants is needed to enhance the prevalence of positive health behaviors, particularly in at-risk populations like teenagers (Leme et al., 2016; Mc Morrow et al., 2017). An unhealthy diet, defined as a high sugar and fat intake with a low intake of fruits and vegetables, is one of the primary causes of non-communicable illness worldwide and is closely linked to early death. Poor food habits are a major contributor to the obesity pandemic (Loef and Walach, 2012; Matthews et al., 2016). The relevance of health and nutrition to one’s Self-Perception may have a significant impact on one’s actions. To assess its influence on eating patterns, other terminology has been employed, such as health interests (Tromp et al., 2005; Sun, 2008). These conceptual frameworks, regardless of nomenclature, emphasize the significance of worries about health, food, and nutrition.

A lot of research has gone into assessing consumer perceptions of food dangers, and the number of such studies has risen in recent decades for a variety of reasons. Highly processed foods, for example, have been widely introduced to the market, but food has also been implicated in several food crises (Leikas et al., 2009; Martinez-Poveda et al., 2009). Risks and issues related to one’s individual lifestyle are frequently overlooked in comparison to societal risks (Nawaz et al., 2020b). Because most individuals believe they have control over their eating habits, perceived health and nutrition hazards may have little influence on their eating habits. However, it appears that among pre- and reproductive-age females, reported concerns about food and health are of greater relevance than among other groups. To begin with, eating habits formed as a youngster may not only follow a woman into adulthood but may also be handed on to her off-spring (Mikkilä et al., 2005; Palfreyman et al., 2014). Second, effective preventative programs for changing the diet of young females (and, by extension, their children) are still required to boost their nutritional status and, as a result, to lower obesity and malnutrition rates (Mikkilä et al., 2005; Palfreyman et al., 2014).

Dissection of perceived health threats result into perceived severity, perceived vulnerability, and fear. These perceptional threats are of the same concern regarding junk food eating behaviors leading to obesity. The adverse repercussions that a person identifies with an event or outcome, such as a cancer diagnosis, are referred to as perceived severity (also defined as the perceived seriousness). These implications might be related to a future occurrence or a present situation, such as a pre-existing health problem. Although perceived severity is a type of negative value and unfavorable polarity, the word appears to get its origins in the Health Belief Model (HBM). The paradigm is based on psychological research and Lewin’s behavioral motivation theory (Luger, 2013). According to Burnes (2019), behavior is determined by two factors: (1) what importance a person places on a certain result, and (2) actual possibility that an individual will succeed in reaching their goal. The key components of ‘expectancy-value’ theories are these two aspects together.

The HBM was created to better understand how preventative and early detection practices are adopted. The paradigm claims that illness severity and perceived vulnerability to disease combine to generate a ‘threat,’ and that threat perception motivates action. According to the paradigm, threat perception drives behavior. Furthermore, signs to action were considered as important to set the process in motion, such as the appearance of symptoms or having a medical appointment (Steckler et al., 2010). Perceived severity could have been utilized as an indicator of obesity due to eating unhealthy junk food. Perceived vulnerability, also known as perceived susceptibility, perceived likelihood, and perceived probability, refers to a person’s perception of the possibility of health hazard or the development of health condition (Gochman and Saucier, 1982).

Perceived vulnerability is an integral component of a threat evaluation process, according to Protection Motivation Theory, implying that people actively participate in assessing their risk. The Precaution Adoption Process greatly broadened the construct by proposing that people go through multiple phases of identifying their vulnerability, ranging from not being aware of the danger to being aware of the threat to eventually admitting that they are personally at risk. Perceived vulnerability could help in defining danger of obesity associated with eating unhealthy or junk foods (Magallares et al., 2015) so, it could lead to be an integral part of our research model.

Fear is also an important factor in eating healthy or unhealthy food which could lead to obesity. When a person is confronted with a threat, they may get fearful. Fear has been linked to increased arousal, negative or unpleasant subjective experiences, and a distinctive facial expression that includes wider eyes and an open mouth. Fearful stimuli have been shown to improve visual perception in studies. In an array of distracter pictures, fear-inducing stimuli are found faster than fear-irrelevant stimuli (Öhman et al., 2001). One of the roles of fear, according to evolutionary perspectives on emotions, is to improve perception (Susskind et al., 2008). This kind of fear has played a significant role in determining the health-related habits. This could also lead to behaviors related to eating healthy or unhealthy junk food leading to obesity.

The prevalence of bad eating habits has been connected to the adoption of a western lifestyle by individuals in emerging countries. A lack of consistency in eating breakfast, a low frequency of fruit and vegetable intake, a proclivity toward increased junk food consumption, and a high frequency of soft drink use are all examples of harmful dietary patterns. Breakfast consumption contributes to positive changes in the hormonal, and neurological processes that regulate intake of food, whereas missing breakfast leads to obesity. Vegetables and fruit, on the other hand, are high in water and fiber and have a low energy density. As a result of the satiating impact of fiber, they defend against obesity by resulting in fewer calories ingested and the displacement of energy-dense meals (Howarth et al., 2001; Rolls et al., 2004; Leidy et al., 2013). Junk food and soft drinks, on the other hand, are high in energy and contain empty calories, which contribute to obesity (Hao et al., 2020). Resultantly, shifting dietary habits, which include increasing intake of more energy-dense foods and decreasing consumption of less energy-dense foods, may be inflicting double damage (Nawaz et al., 2020a).

According to the existing research on knowledge sharing, managers who share their information have beneficial effects on personal productivity and individual job performance, team creativity and innovation, and organizational financial performance. Although information concealing and other comparable notions, such as knowledge sharing and knowledge hoarding, are said to have some similarities or overlap, knowledge hiding differs from these ideas in at least two respects (Dong et al., 2016). Although no research in so far has been conducted on knowledge hiding about products but there is a scope of finding the role of product knowledge hiding on different health behaviors and the consequences of those behavior. So, moderating role of product knowledge hiding was suggested in our study context.

Dietary behavior is changing dramatically on a global scale, and it is connected to the “New World Syndrome,” in which emerging nations change their habits and lifestyles to a junk food-based sedentary lifestyle, often in slavish imitation of the West, and fast-food firms’ relentless marketing. Obesity has been recognized as one of the first waves of this New World Syndrome, followed by a slew of chronic ailments that are wreaking havoc on emerging nations’ socioeconomic and public health systems (Magallares et al., 2015). These have now reached worldwide epidemic proportions and should be considered today’s most pressing public health issue. These things produce several research questions such as how perceived health threats could lead to obesity? how junk food eating behavior could mediate the relationship of perceived health threats with obesity? What regulations product knowledge hiding could impose on the mediating relationship of junk food eating behavior with obesity. To address these questions, following research was designed to analyze the impact of perceived health threats such as perceived severity, perceived vulnerability and fear on obesity, evaluate the mediating role of junk food eating behavior between the relationship of perceived health threats with obesity and exploring the moderating role of product knowledge hiding between mediating junk food eating behavior and obesity problems.

## Review of Literature and Hypotheses Development

The factors of perception regarding eating unhealthy or junk food leading to obesity are based on some theories of expectancy value. Such a theory which provided basis for devising the relationships of perceived vulnerability, perceived severity and fear of eating junk food habits leading to obesity is explained below.

### Protection Motivation Theory

The PMT model was created to explain how fear appeals affect health attitudes and behaviors (Rogers, 1975). Fear-inducing communications have a significant influence on behavior selection. Improvements in the perceived level of fear frequently led to increases in acceptability of the adaptive control action or intention, according to a meta-analysis of studies of fear-arousing messages published between 1953 and 1980 (Sutton and Hallett, 1988). Increases in perceived response efficacy also enhanced the likelihood of choosing the ability to respond. Theory has been used to a varied range of themes, including areas of interest outside health-related difficulties, according to a detailed narrative assessment of the literature and research on theory (Prentice-Dunn et al., 1997). Theory has been expanded to environmental concerns, prevention of accidents, safeguarding others, and political issues in addition to health promotion and illness prevention.

Protection motivation theory is a commonly used paradigm for understanding how people react to stimuli that alert them to a possible hazard. Such stimuli include fear signals that allow people to take precautions or avoid from taking actions that could damage them or others. This idea belongs to the category of expectation value theories, which claim that certain attitudes or beliefs will lead to specific behaviors. People discuss possible responses through a threat better coping evaluation process. The threat evaluation method includes determining the danger’s severity as well as the possibility (or vulnerability) of the threat occurring. The efficacy of the reaction, the difficulty of enacting the response, and the perceived Self-Efficacy of executing the coping response are all factors considered throughout the coping assessment process. When the danger appraisal outweighs the coping appraisal, the result is a maladaptive reaction (Shillair, 2020).

Rogers examined the literature on theory and discovered that theory and its elements were well supported. Their finding, however, was predicated on a narrative examination. The degree of the influence of the protection motivation theory aspects might be assessed with a better quantitative grasp of the model variables. Fear is assessed in PMT to forecast and motivate protective responses, as well as to explain the cognitive processes involved in danger and coping assessments (Sadeghi et al., 2019). Threat and coping assessments can result in adaptive or maladaptive reactions, both of which represent health risks (Rogers, 1975). The following elements influence threat evaluation in PMT: (1) perceived severity, (2) perceived vulnerability, and (3) perceived benefits. As a result, there is a larger motivation for engaging in health-promoting actions when the perceived severity and vulnerability are high and the perceived benefits are low. Fear is a mediator between perceived vulnerability, severity, and threat evaluation in general.

Resultantly, if someone feels susceptible to a major health hazard, their anxiety level rises, and they are more driven to engage in preventive/protective action (Sadique et al., 2007; Watkins et al., 2007; Sharifirad et al., 2014; Ling et al., 2019). To establish protective motivation, coping and threat evaluation processes are combined. It is suggested that PMT may properly predict whether or not protective behaviors would be adopted (Lowe et al., 2000; Jiang et al., 2009; Zare Sakhvidi et al., 2015; Babazadeh et al., 2017). PMT has been used to explore a variety of behaviors, including influenza vaccine injection, H1N1 pandemic prevention, cancer prevention and sun protection behaviors, SARS prevention behaviors, and communicable diseases and skin cancer prevention behaviors (Ezati Rad et al., 2021). Based on this theory and its components, our research was designed for analysis of perceived severity, perceived vulnerability, and fear about eating junk food leading to obesity.

### Relationship of Perceived Health Threats With Junk Food Eating Behavior

Changing a behavior, adopting a protective behavior, or doing both. One can adopt a new behavior, change an old one, or participate in behaviors that blend new and modified features while altering behavior (Rosenstock, 1974; Witte and Allen, 2000). Danger assessments are based on one’s impression of the threat’s severity as well as one’s vulnerability to the threat. The severity of a threat is determined by severity, but vulnerability is determined by the likelihood of being in danger (Neuwirth et al., 2000; Umeh, 2003; McMath and Prentice-Dunn, 2005). A person is more likely to adjust to a situation when they have a strong conviction in the threat’s seriousness and personal vulnerability. Information that is potentially dangerous might originate from a variety of places, including previous experiences (Umeh, 2004). Prior correlation research addressing the association between perceived danger and healthy eating behavior have frequently recruited certain at-risk populations, such as those who are more susceptible to memory loss and cardiovascular disease (Cox et al., 2004; McKinley, 2009). Given that obesity has surpassed cigarette smoking and alcohol consumption as the leading cause of death in the United States, the threat of obesity is likely to enter the minds of a much larger populace.

Furthermore, earlier research that used threat measures to explain eating behavior did not look at fruit and vegetable consumption. Different studies have frequently assessed harmful fat intake without additionally examining fruit and vegetable intake. People are often presented with several options when determining what meals to eat, and models should account for these decisions (Baranowski et al., 2003). Only assessing the desire to eat harmful fat items yielded inconsistent findings, especially in terms of severity and susceptibility. In Australia, for example, a community at risk for cardiovascular disease was studied. Reaction efficacy (conceptions of a suggested initiative’s effectiveness) and Self-Efficacy (perceptions of one’s ability to successfully execute actions that will lead to a desired outcome) were found to be positively associated with low–fat diet intentions, whereas severity and vulnerability were not (Bandura, 1982; Plotnikoff and Higginbotham, 1995). One of the study’s aims was to use a multidimensional measure of healthy eating behavior that included both intake of fruits and vegetables as well as items with less harmful fat. More significantly, previous research on severity and susceptibility has indicated that both of these factors adversely influence healthy lifestyle goals, contrary to what PMT suggests (Plotnikoff and Higginbotham, 2002).

Increased vulnerability was shown to be adversely connected to workout goals. Recently, several researchers conducted a study on teenage perceptions of the risk for cardiovascular disease. Interestingly, greater assessments of the severity of cardiovascular disease indicated the desire to consume fatty meals. Perceived danger can lead to maladaptive or defensive behaviors at low levels of effectiveness (Umeh, 2003). As a result, some of the Umeh research participants may have had low esteem or behavioral efficacy, and hence behaved defensively to perceived threat. Unfortunately, neither Plotnikoff and Higginbotham’s nor Umeh’s studies directly tested this. Overall, it is yet unknown how threat perception will influence healthy eating habits. Even though many students are neither obese or overweight and so may get away with a bad diet, adopting good eating habits in college may inculcate habits that will help avoid a range of obesity concerns in the future. Obesity, as previously said, has become a more serious health issue than cigarette smoking or alcohol consumption. Additionally, one might minimize their alcohol intake by avoiding specific social occasions. Students, on the other hand, are faced with a range of dietary and nutritional choices on a regular basis (Huang et al., 2003). These challenging choices, together with findings indicating over 20% of college students are overweight or obese, imply that severity and susceptibility are two powerful factors that may influence college students’ eating decisions.

However, little is known about the relationship between perceived obesity dangers and eating habits. Previous study findings give little reason to believe that a favorable association between threat perception characteristics and healthy eating behavior may be predicted. If a negative relationship between these factors is discovered, future researchers may look at ways to reduce weight-related anxiety. As a result, the study’s initial purpose was to look at the primary effects of perceived severity, perceived vulnerability, and fear on junk food consumption. In this regard the following hypotheses were formulated to analyze the relationship of these with the junk food eating behavior leading to obesity.

*H*_*1*_: Perceived severity has a negative effect on junk food eating behavior.

*H*_*2*_: Perceived vulnerability has a negative effect on junk food eating behavior.

*H*_*3*_: Fear has a negative effect on junk food eating behavior.

### Relationship of Junk Food Eating Behavior With Obesity

A lot of research in this regard has been carried out previously in different perspectives. There is a general notion that eating unhealthy or junk food leads to obesity which is considered as a disease in some contexts (Huang et al., 2003; Bayol et al., 2007; Yaniv et al., 2009; Datar and Nicosia, 2012; Hemmingsson, 2018; Robinson et al., 2021). The prevalence of bad eating habits has been connected to the adoption of a western lifestyle by individuals in emerging countries. A lack of consistency in eating breakfast, a low frequency of fruit and vegetable intake a proclivity toward increased junk food consumption, and a high prevalence of soft drink use are all examples of harmful dietary patterns. Breakfast consumption contributes to positive changes in the appetitive, hormonal, and neurological signals that regulate food intake, whereas missing breakfast leads to obesity. Fruits and vegetables, on the other hand, are high in water and fiber that have a low energy density (Huang et al., 2003; Bayol et al., 2007; Sun, 2008; Yaniv et al., 2009; Datar and Nicosia, 2012; Loef and Walach, 2012; Mc Morrow et al., 2017; Hemmingsson, 2018; Robinson et al., 2021). As a result of the satiating impact of fiber, they defend against obesity by resulting in fewer calories ingested and the displacement of energy-dense meals. Junk food and soft drinks, on the other hand, are high in energy and contain empty calories, which contribute to obesity. Thus, the changing dietary patterns, which simultaneously include a higher consumption of the more energy-dense food and a lower consumption of the less energy-dense food, could be causing double harm (Faizi et al., 2018). These supported literatures and the pre-existing relationship allowed us to find the impact of junk food eating behavior on obesity and we devised the following hypothesis.

*H*_*4*_: Junk food eating behavior has a positive effect on obesity.

### Mediating Role of Junk Food Eating Behavior

There is a general notion that eating unhealthy or junk food leads to obesity which is considered a disease in some of the contexts (Huang et al., 2003; Bayol et al., 2007; Yaniv et al., 2009; Datar and Nicosia, 2012; Hemmingsson, 2018; Robinson et al., 2021). The prevalence of bad eating habits has been connected to the adoption of a western lifestyle by individuals in emerging countries. A lack of consistency in eating breakfast, a low frequency of fruit and vegetable intake, a proclivity toward increased junk food consumption, and a high prevalence of soft drink use are all examples of harmful dietary patterns. Breakfast consumption contributes to positive changes in the appetitive, hormonal, and neurological signals that regulate food intake, whereas missing breakfast leads to obesity. Fruits and vegetables, on the other hand, are high in water and fiber and have a low energy density (Huang et al., 2003; Bayol et al., 2007; Sun, 2008; Yaniv et al., 2009; Datar and Nicosia, 2012; Loef and Walach, 2012; Mc Morrow et al., 2017; Hemmingsson, 2018; Robinson et al., 2021).

Although, it is evident from the literature that certain behaviors could mediate the relationships between vulnerability, obesity, severity and fear but no prior research has focused on evaluating the mediating role of junk food eating behavior. This leads to utilizing this behavior as mediator in our context of the study. It was suggested by looking into the habitual behaviors playing a mediating role between change in symptoms of depression and food related behaviors (Owens et al., 2021). Certain attitudes have been reported in past to be mediated by some behaviors for eating healthy. For an instance, diet relationships at educational level in directing attitudes toward healthy eating have been studied in past and offered some significant results (Lê et al., 2013). It was further suggested by Wu et al. (2019), that dietary behaviors whether eating junk or healthy food could mediate the relationships between obesity and food insecurity. This research provided an insight about mediating role of behavior of junk food eating. Keeping in view the novelty of this behavior as a mediator between perceived severity and obesity, perceived vulnerability and obesity and fear and obesity, we hypothesized the following for analysis.

*H*_*5*_: Junk food eating behavior mediates the relationship of perceived severity and obesity.

*H*_*6*_: Junk food eating behavior mediates the relationship of perceived vulnerability and obesity.

*H*_*7*_: Junk food eating behavior mediates the relationship of fear and obesity.

### Moderating Role of Product Knowledge Hiding

Researchers discovered that, while rationalizing their actions, knowledge hiders foresaw impaired relationships (Connelly and Zweig, 2014). There are other grounds to suspect that managers from different companies might purposefully conceal information during commercial interactions, isolating companies. For example, a providing manager may be hesitant to share information with the purchasing manager because he does not completely trust the latter (Hernaus et al., 2018). Lack of interpersonal interactions is one aspect that might contribute to a lack of trust. Managers from different organizations may also suppress knowledge during their supply chain interactions if they feel that sharing, would have a negative impact on them or they will get no personal advantage, if they share it with their supply chain counterpart. Managers can also hide knowledge if their organization’s culture encourages it.

Employees may act in ways that are counter to their role expectations but yet adhering to company rules in order to maintain their employment. Another explanation might be possible, if managers from different companies dislike one other during business interactions, resulting in them withholding information from one another. According to the researchers, managers’ capacity to explore and develop new ideas, execute change, apply new information, or improve procedures to increase personal and corporate performance and understanding is harmed by knowledge concealing (Arain et al., 2020). The literature on the buyer–supplier interaction in the supply chain is few and elusive. In other words, it does not explain why purchasing and supplying business executives keep information from one other throughout supply chain interactions (Avotra et al., 2021; Yingfei et al., 2021; Dar et al., 2022). Moderating role of knowledge hiding and hiders is studied less in past, e.g., (Chatterjee et al., 2021) but it provided us to analyze and find deep into the said moderation between junk food eating behaviors and the outcome of it, “obesity.” So we hypothesized the following.

*H*_*8*_: Product knowledge hiding moderates the effect of junk food eating behavior on obesity.

**Figure 1:**
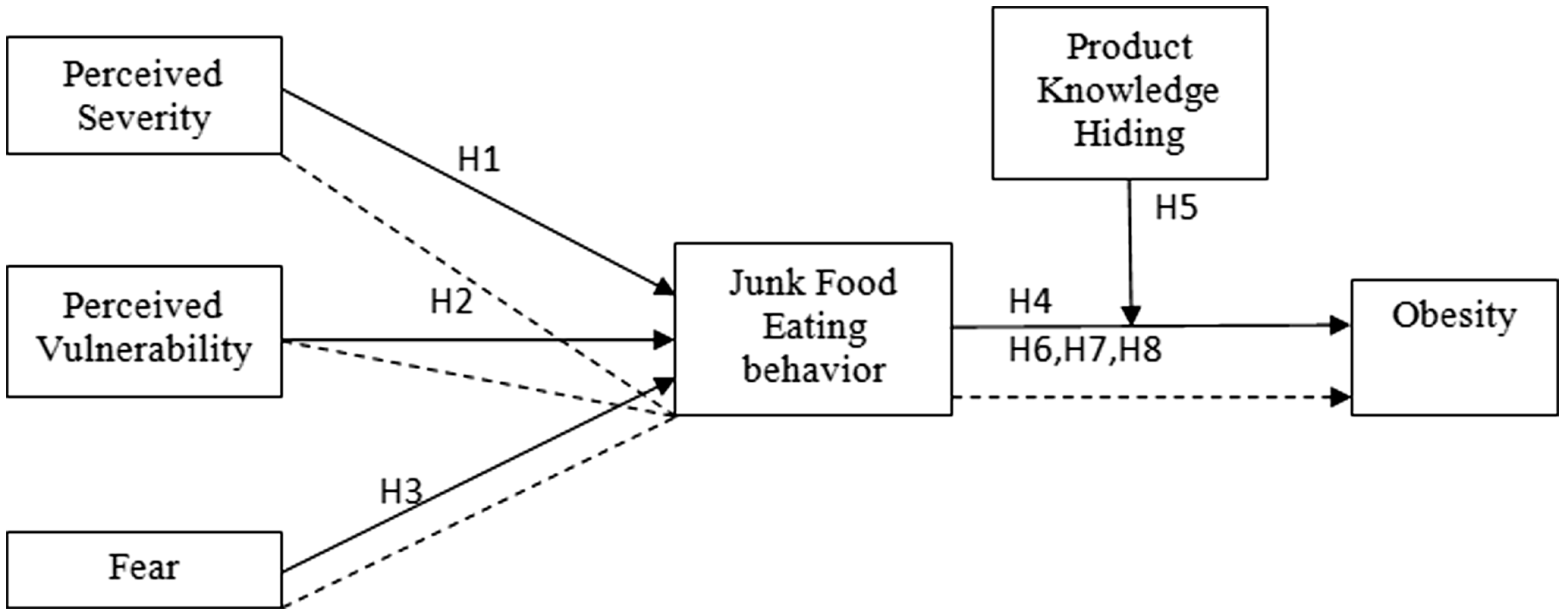
Conceptual model.

This research is based on the following conceptual model (see Figure 1) for the analysis of perceived health threats on developing junk food eating behavior leading to obesity with a moderating effects of product knowledge hiding.

## Methodology

This study is based on the quantitative analysis where the effects of perceived severity, perceived vulnerability and fear are checked on the obesity of kids through junk food eating behavior. The moderating role of product knowledge hiding had been measured on the relationship of junk food eating behavior and obesity of teenagers. The population of the study were the kids and adolescents in their teens who are more involved in junk food eating behaviors (Boylan et al., 2017). The total number of kids and adolescents reached out for the data collection were 400 however the usable responses received were 228. The sampling method used in this study is purposive sampling as getting track of all kids and adolescents in China is not easy (Saha et al., 2021). Therefore, the kids and adolescents were reached on purpose for data collection. This was an adequate group for obesity to be checked as they are among the group who are highly involved in junk food eating. The data collection method used through survey questions, and the participants were informed in advance for their services to be rendered for the data collection purpose. The guardians of the respondents were also informed to give consent if they want to participate or not and, the anonymity of the respondents was ensured. The questionnaires were distributed individually and were given time to fill it out and it was Self-Administered to avoid any ambiguities. Questionnaires were the best technique to be used for data collection as it documents the responses and gives respondents option to the severity of the agreement of the respondents. The data was statistically treated with the help of Smart-PLS software for the structural equation modeling because it measures the relationships simultaneously for all the variables.

### Instrument Development

The instrument used for data collection is the questionnaire-based survey. There were 31 items in total in the survey which were adapted from the previous studies as there were found most appropriate in the literature that represent the variables of the present study. The 6-items scale for the junk food eating behavior has been adapted from (Schlundt et al., 2003). It addressed the eating behavior patterns and the involvement of the respondents in junk food. This questionnaire was especially designed in understanding the junk food eating behaviors. The measurement scale for perceived severity and perceived vulnerability were consisted of 4-itesms each, while the measurement scale for the variable fear contained three items. These three scales have been adopted from (Pang et al., 2021) who had measured the role of these three variables in the organic food patterns of the respondents. Further, the variable of obesity is measured with a 10-item scale developed by Schlundt et al. (2003). Additionally, the variable of product knowledge hiding had been adapted and modified according to the study taking the playing dumb perspective of the knowledge hiding developed by Connelly et al. (2012). It fits the product knowledge hiding variable of the present study in the best appropriate way. It consisted of 4-items (sample items: “Pretended that I do not know which information is related to product,” and “explains that, I have no knowledge about the constituents of this product,” etc.). The previously established scales were found most appropriated addressing the constructs of variables used in the present study. Furthermore, the scales used in this study were validated with discriminant validity and convergent validities along with the Cronbach *α* and composite reliabilities. The average variance extracted had also been used to verify the validity of the scales used. The details for reliabilities and validities are given in the following sections.

### Demographics Details

The first section of the questionnaire was consisted of the demographic information for the respondents. The population of the study were the kids and the teenagers therefore, the age was divided into two main categories only, i.e., below 12 and above 12. The education was divided into three categories of grades 5–10, 11–12, and above 12 because the respondents of the study fall in kids and teen categories. The details of the demographic analysis are given in table analysis are given in Table 1.

**Table 1:** Demographics analysis.

Demographics	Frequency	Percentage
**Gender**		
Male	152	66.66
Female	76	33.33
**Age**		
Below 12 years	105	46.05
Above 12 years	123	53.94
**Education**		
Grade 5–10	72	31.57
Grade 11–12	111	48.68
Above grade 12	45	19.73

*N* = 228.

## Data Analysis and Results

Data of the study were analyzed with the software Smart-PLS for the partial least square structural equation modeling. In this software, the relationships of the independent variables (i.e., perceived severity, perceived vulnerability and fear) are measured with the junk food eating behavior and its consequence in the form of obesity. The moderating effect of product knowledge hiding has been checked for the effect of junk food eating behavior on obesity of the kids and adolescents. Smart-PLS measures the model in two phases, i.e., measurement mode and the structural mode. The measurement model gives the validity and reliability of the scales, while structural model checks the acceptance or rejection of the hypothesis based on the statistics produced like *r*-square, *f*-square, *t*-statistics, *p*-values, and the *β* values.

### Measurement Model

**Figure 2:**
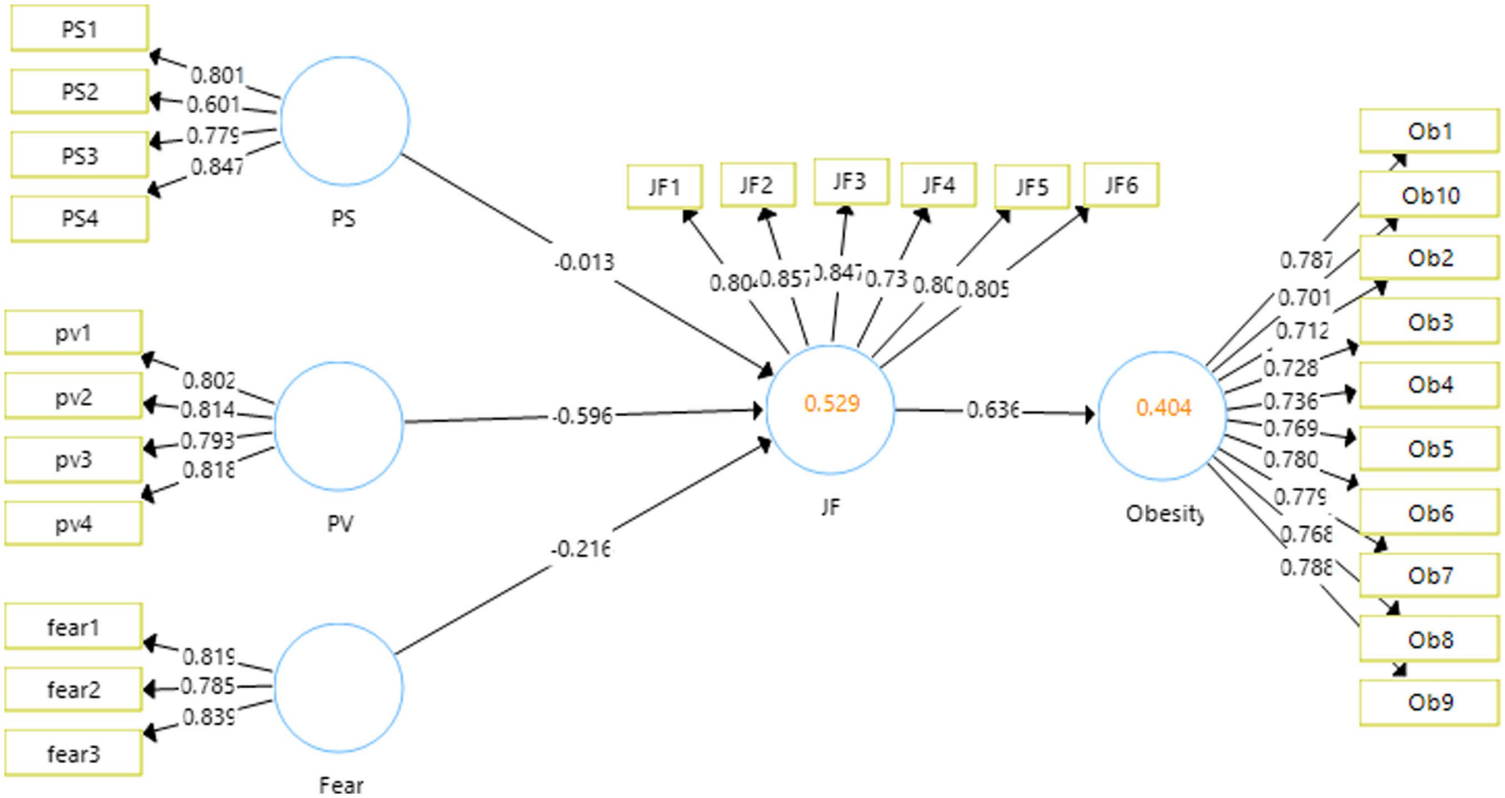
Output of measurement model. PS, perceived severity; PV, perceived vulnerability; JF, junk food eating behavior.

The measurement model reports the validities, reliabilities, and *β* values of the variables. The output for the measurement model has been represented in the Figure 2.

The statistics for the factor loadings, reliabilities and average variance extracted are reported in the Table 2. The minimum threshold for the reliabilities and the factor loadings of the items has been reported as 0.7 (Henseler et al., 2015). All the values obtained in this study are above this threshold, thus meeting the criteria of convergent validities of the scale. The minimum values obtained for factor loadings are 0.701, rest of the values are above this thus making the scale convergent valid. The values for outer variance inflation factor of the items used in this study to check the collinearity among the items, however, all are found significant as they all are less than the threshold mentioned in the literature (Grewal et al., 2004). Similarly, the reliabilities obtained in this study for the variable, minimum statistics for Cronbach alpha reliability is 0.752 and for composite reliability is 0.846, which are well above the prescribed threshold in the literature. Further, the average variance extracted is reported to be above 0.5 (Di Marco et al., 2018). The minimum value obtained in this study for AVE is 0.565.

**Table 2:** Factor loadings, reliabilities, VIF, and AVE.

Variables	Factor loadings		VIF	Cronbach *α*	Composite reliability	AVE
Junk food	JF1	0.804	2.851	0.895	0.920	0.656
	JF2	0.857	3.760			
	JF3	0.847	2.990			
	JF4	0.738	2.153			
	JF5	0.805	2.617			
	JF6	0.805	3.186			
Obesity	Ob1	0.768	4.587	0.919	0.928	0.565
	Ob2	0.702	1.684			
	Ob3	0.701	2.948			
	Ob4	0.707	4.383			
	Ob5	0.712	4.289			
	Ob6	0.749	4.127			
	Ob7	0.796	2.519			
	Ob8	0.800	2.832			
	Ob9	0.785	2.487			
	Ob10	0.805	2.643			
Product knowledge hiding	PKH1	0.836	2.051	0.855	0.902	0.698
	PKH2	0.875	2.349			
	PKH3	0.873	2.352			
	PKH4	0.752	1.640			
Perceived severity	PS1	0.801	1.601	0.755	0.846	0.582
	PS2	0.701	1.248			
	PS3	0.779	1.713			
	PS4	0.847	1.924			
Fear	fear1	0.819	1.577	0.752	0.856	0.664
	fear2	0.785	1.575			
	fear3	0.839	1.412			
Perceived vulnerability	pv1	0.802	1.423	0.826	0.882	0.651
	pv2	0.814	2.038			
	pv3	0.793	1.986			
	pv4	0.818	1.936			

Another measure used for validity checking is the heterotrait-monotrait (HTMT) ratio and the Fornell and Larcker Criteria. These are the validity measures for discriminant validities of the scales. Present study has also employed these two validity tests to measure the discriminant validity of the scaled adapted in this study. The results of the HTMT ration are supposed to show values less than 0.9 (Franke and Sarstedt, 2019), which are reported in Table 3. The reported values for HTMT ration are below the mentioned cut off criteria. The maximum value obtained for HTMT ratio for this study is 0.808.

**Table 3:** HTMT ratio.

	Fear	JF	Obesity	PKH	PS	PV
Fear						
JF	0.591					
Obesity	0.718	0.664				
PKH	0.501	0.772	0.703			
PS	0.554	0.403	0.808	0.376		
PV	0.538	0.774	0.683	0.610	0.470	

JF, junk food eating behavior; PS, perceived severity; PV, perceived vulnerability; PKH, product knowledge hiding.

Similarly, another statistical test used is Fornell and Larcker Criteria. The values obtained should show the highest value at the top of the column for each variable (Franke and Sarstedt, 2019). The present study meets this criteria, as the highest values lie at the top for each variable. The results can be seen in Table 4.

**Table 4:** Fronell and Larcker Criteria.

	Fear	JF	Obesity	PKH	PS	PV
Fear	0.815					
JF	−0.496	0.810				
Obesity	−0.582	0.640	0.752			
PKH	−0.410	0.681	0.688	0.836		
PS	0.438	−0.333	−0.615	−0.309	0.763	
PV	0.461	−0.701	−0.660	−0.543	0.378	0.807

JF, junk food eating behavior; PS, perceived severity; PV, perceived vulnerability; PKH, product knowledge hiding.

Moreover, the adjusted *r*-square reported for the study are 52.9% for junk food eating behavior and 40.4% for the obesity variable, that shows the variables used in this study for the prediction of junk food eating and obesity are relevant to these variables. Similarly the inner VIF values have also been found within the significant range, i.e., 3.3 (Grewal et al., 2004).

### Structural Model

**Figure 3:**
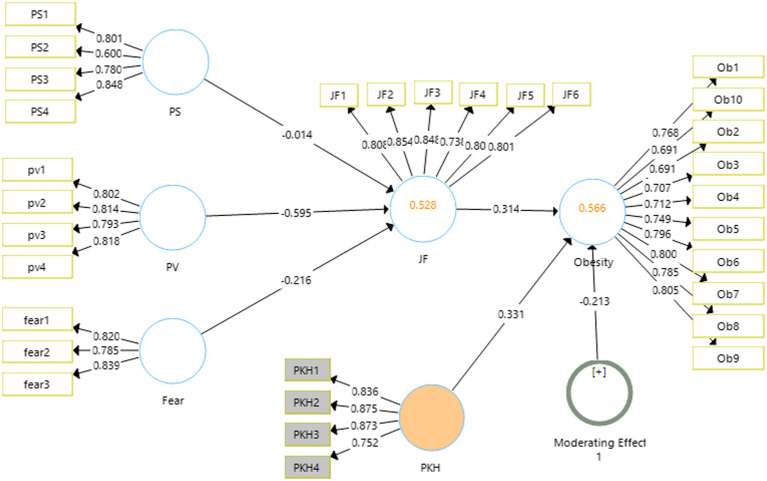
Output of measurement model with moderation. SD, standard deviation; JF, junk food eating behavior; PS, perceived severity; PV, perceived vulnerability; PKH, product knowledge hiding.

The output for the structural model is shown in Figure 3. It shows the strength of the relationship of the independent variables with the dependent variables along with significance.

The Table 5 of the study reports the mean sample and original sample with the *t*-stats and *p*-values obtained for each hypothesis of the study. The results obtained go along the hypotheses generated in this study. Perceived severity, perceived vulnerability and fear show the negative relationships with the junk food eating behavior which is justified with the results obtained. Similarly, the junk food eating behavior shows a positive and significant relationship with obesity among the kids.

**Table 5:** Direct effects.

Paths	*H*	*O*	*M*	SD	*t*-statistics	*p*-value	*R* ^2^	*F* ^2^	VIF	Results
PS ➔ JF	*H*_1_	−0.013	−0.018	0.057	0.261	0.794			1.301	Rejected
PV ➔ JF	*H*_2_	−0.597	−0.594	0.054	11.032	0.000^***^	0.529	0.566	1.335	Accepted
Fear ➔ JF	*H*_3_	−0.216	−0.220	0.057	3.987	0.000^***^		0.070	1.416	Accepted
JF ➔ Obesity	*H*_4_	0.316	0.318	0.075	15.682	0.000^***^	0.404	0.679	1.000	Accepted

*** *p* < 0.001.

H, hypothesis; O, original sample; M, sample mean; SD, standard deviation; JF, junk food eating behavior; PS, perceived severity; PV, perceived vulnerability; PKH, product knowledge hiding.

The first hypothesis of the study could not find significant outcome owing the effect of perceived severity on junk food eating (*H*_1_: *t*-stats = 0.261, *p* > 0.05), hence the first hypothesis of the study is rejected. The second hypothesis (*H*_2_: Perceived vulnerability has a negative effect on junk food eating behavior; *t*-stats = 11.032, *p* < 0.001, *β* = −0.597) is accepted with a negative effect on junk food eating behavior. Regarding third hypothesis of the study (*H*_3_: Fear has a negative effect on junk food eating behavior, *t*-stats = 3.987, *p* < 0.000, *β* = −0.216) has shown significant negative effects on the junk food eating behaviors. Similarly, the fourth hypothesis (*H*_4_: Junk food eating behavior has a positive effect on obesity, *t*-stats = 15.682, *p* < 0.001, *β* = 0.316) is accepted with junk food eating behavior showing a positive significant effect on the obesity of the kids and adolescents (Table 6).

**Table 6:** Indirect effects.

Paths	*H*	*O*	*M*	SD	*t*-statistics	*p*-value	Results
Fear ➔ JF ➔ Obesity	*H*_5_	−0.137	−0.140	0.028	3.583	0.000^***^	Accepted
PS ➔ JF ➔ Obesity	*H*_6_	−0.009	−0.014	0.019	0.256	0.830	Rejected
PV ➔ JF ➔ Obesity	*H*_7_	−0.378	−0.381	0.046	9.200	0.000^***^	Accepted

*** *p* < 0.001.

H, hypothesis; O, original sample; M, sample mean; SD, standard deviation; JF, junk food eating behavior; PS, perceived severity; PV, perceived vulnerability; PKH, product knowledge hiding.

**Figure 4:**
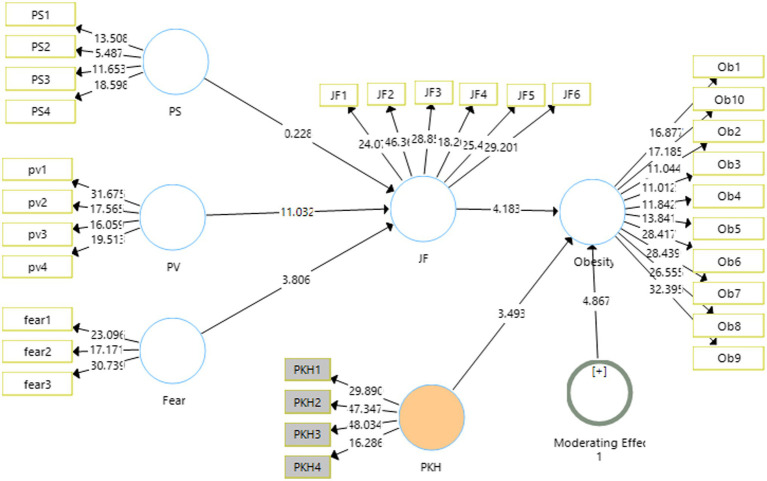
Output of structural model with moderation. PS, perceived severity; PV, perceived vulnerability; JF, junk food eating behavior; PKH, product knowledge hiding.

Regarding the indirect effects of the study, two relationships have been found significant while one could not find any significance in this study. The mediation of junk food between fear and obesity have been found to have negative significant effect, *t*-stats 3.583, *p* < 0.05, *β* = −0.137. Similarly perceived vulnerability also found to have significant negative relationship with obesity in the presence of junk food *t*-stats 9.20, *p* < 0.001, *β* = −0.378 (Figure 4).

Product knowledge hiding has been used as the moderating variable in this study that is proposed to moderate the relationship between junk food eating behavior and obesity. Before measuring the significance of the hypothesis of moderations, the measurement model was again checked for reliability and validity of the scale. These all have been found significant; the reliabilities had been reported as 0.895 for junk food eating behavior, 0.752 for the variable fear, 0.919 for obesity, perceived vulnerability 0.826 and 0.755 for perceived severity. Similarly, the validities had also been under the prescribed values mentioned (Table 7).

**Table 7:** Moderating effects.

Paths	*H*	*O*	*M*	SD	*t*-statistics	*p*-value	*F* ^2^	Results
PKH × Junk food ➔ obesity	*H*_8_	−0.213	−0.225	0.044	4.867	0.000^***^	0.088	Accepted
PKH ➔ Obesity		0.330	0.319	0.094	3.493	0.001^**^	0.106	

*** *p* < 0.001;

** *p* < 0.05.

H, hypothesis; O, original sample; M, sample mean; SD, standard deviation; PKH, product knowledge hiding.

Moving to the fifth hypothesis (*H*_5_: Product knowledge hiding moderates the effect of junk food eating behavior on obesity, *t*-stats 4.487, *p* < 0.001, *β* = −0.213) showing higher awareness of the product knowledge hiding, weaker will be the relationship of junk food eating behavior and obesity among kids.

## Discussion

There was a strong need of conducting the research on perceived health related threats for developing junk food eating behaviors which ultimately leads to obesity. Our research focused on the developed model discussed earlier in which constituents of the perceived threats were analyzed for their impact on developing junk food eating behaviors. Such behaviors were setting on the renowned obesity considered as disease in previous literature. Perceived severity, perceived vulnerability and fear were the elements drawn from the perceived health threats suggested by HBM by Gochman and Saucier (1982) and supported by protection motivation theory by Rogers (1975). The analysis was conducted through Smart-PLS which yielded into mixed results regarding associations of these factors which ultimately leads to obesity.

Our first hypothesis which was about perceived severity having a negative effect on junk food eating behavior. This hypothesis was rejected, and the reason of its rejection was the strong and positive association of perceived severity with eating junk food behaviors. These results are in accordance to some researchers such as (Tavassoli et al., 2018). It is obvious that, prior to taking any action to conduct a certain behavior, persons’ knowledge and awareness of the issue should be evaluated, and they should be educated, if their understanding of the behavior and its many elements is lacking. They should then be taught how to do that activity. In that instance, it is expected of the individual to excel at a certain behavior. According to these findings, perceived severity necessitates greater attention since low perceived severity might lower accuracy in completing the action, leading to people eating more junk food.

The second hypothesis which was about impact of perceived vulnerability on developing junk food eating behaviors was accepted as perceived vulnerability under the umbrella of perceived health threat put a negative impact on eating junk food and developing this behavior. These results are supported by the previous studies of various researchers in different perspectives such as (Umeh, 2003, 2004). Our next hypothesis was about perceived fear of eating junk food behavior. This proposition was also accepted as fear had negative association with developing junk food eating behavior. The fear of getting obese led to the production of such results in which it is considered as a disease. This fact is also supported by the development of not eating junk food behaviors in the fitness freaks. Similar findings were obtained by previous researchers in different contexts such as during pandemic (Robinson et al., 2021).

The fourth hypothesis was about eating junk food behaviors which lead to obesity was supported by many researchers of the past with the reasoning of development of such behavior would resultantly set on the obesity (Huang et al., 2003). This is established from decades that behavior of eating unhealthy or the junk food leads to the obesity. so, our hypothesis results were in accordance to such previous researches. The fifth, sixth and seventh hypothesis responded to the mediating role of junk food eating behavior as a mediator between perceived health threats such as perceived severity, perceived vulnerability, fear and obesity. The results indicated that these relationships could be aided by junk food eating behaviors. Although, it is evident from the literature that certain behaviors could mediate the relationships between vulnerability, obesity, severity and fear but no prior research has focused on evaluating the mediating role of junk food eating behavior. This leads to utilizing this behavior as mediator in our context of the study. It was suggested by looking into the habitual behaviors playing a mediating role between change in symptoms of depression and food related behaviors (Owens et al., 2021).

Certain attitudes have been reported in past to be mediated by some behaviors for eating healthy. For an instance, diet relationships at educational level in directing attitudes toward healthy eating have been studied in past and offered some significant results (Lê et al., 2013). It was further suggested by Wu et al. (2019), that dietary behaviors whether eating junk or healthy food could mediate the relationships between obesity and food insecurity. This research provided an insight about mediating role of behavior of junk food eating. Therefore, mediated the direct effects of perceived health threats on obesity. Our last hypothesis was also accepted and yielded significant results and hiding information regarding the products significantly moderates or regulates the association between eating junk food behaviors and the setting of obesity resultantly. These associations are strongly influenced by the product knowledge hiding. As knowledge hiding regarding products would put the consumers on unconsciousness that what they are eating and what would it impact on their health. Such moderating roles of knowledge hiding have been partially studied before such as moderating role of knowledge hiders in different perspective was studied by Hemmingsson (2018). This study provides a handful insight on developing healthy eating behaviors so, it could lead to healthy lives.

### Theoretical Contribution

The present study makes following theoretical contributions to the literature. (1) To the best of our knowledge, this is among the initial studies that check the role of perceived severity, perceived vulnerability, and fear in affecting the junk food eating behaviors. It has shown that the awareness can create the cautious behavior among the users regarding junk foods. (2) The study has estimated that the awareness of the perceived threats creates the negative relationship with the junk food eating that detains the kids and adolescents from eating junk foods. (3) This is already a proven fact that the use of junk food causes obesity among the users. However, the awareness of the product knowledge hiding from the manufacturers end has a negative impact on the positive relationship of junk food eating behavior and obesity. When there is less awareness of product knowledge hiding, more obesity will be seen due to junk food.

### Managerial Implications

There are certain managerial implications of the present study. (1) General public should be made aware of the product contents and the warnings for any undesirable results of using the junk food eating. (2) There lies the responsibility of the manufacturing firms that they should not hide any knowledge about the contents and products moved to the market so the consumers should be aware of the dangers associated and the products used. (3) Obesity being one of the major health issues globally, it is important to make kids aware how to read the information written on the product packages and how it can be helpful for them to understand the risks associated with it.

### Future Research Direction and Limitation

This study has few limitations as well. First of all, it is a cross sectional study, a panel data analysis is required to study if the awareness about the junk food eating behaviors is made available, does this lessens the cases of obesity. Second, it is conducted in China, where less obesity is seen due to healthy eating behaviors, it should be conducted in United States or other Western countries where obesity is a genuine issue. Third, the study has not considered the warning labels on the products information, role of these warning labels can be seen in future, how it affects the junk food eating behaviors among kids. Another limitation of the study is that it has used purposive sampling with kids and adolescents as population. Future study should be conducted taking different age groups including adults as well to know the trend of junk food eating behaviors among them.

## Conclusion

Obesity has become a global issue since the junk foods have been brought to the market. These foods are presented in such an attractive way that kids cannot resist it. However, the manufacturing companies have been hiding certain contents of the products that are major factors for the obesity in the junk food users. Therefore, this study has attempted in measuring the role of junk food eating behavior in obesity among kids and adolescents. However, the role of perceived threats in the form of perceived severity for the use of product, perceived vulnerability of the products and fear of using the product containing obesity triggering contents have been found to have negative impact on the junk food eating behavior. This shows if adequate awareness is given and the perceived threats are mentioned to the kids than it will not engage in the junk food eating behaviors. Furthermore, the awareness of product knowledge hiding has been found to have significant moderation on the relationship of junk food eating behavior and obesity.

## Data Availability Statement

The original contributions presented in the study are included in the article/supplementary material, further inquiries can be directed to the corresponding author.

## Ethics Statement

The studies involving human participants were reviewed and approved by Xian University of Architecture & Technology (XUAT), China. The patients/participants provided their written informed consent to participate in this study. The study was conducted in accordance with the Declaration of Helsinki.

## Author Contributions

YL and XL: writing the draft and conceptualization. TZ, HG, and CS: data collection and funds acquisition. All authors contributed to the article and approved the submitted version.

## Funding

This study was supported by the General project of the National Social Science Fund (20BTY072) and the Education Department of the Shaanxi Provincial Government (18JK0423).

## Conflict of Interest

The authors declare that the research was conducted in the absence of any commercial or financial relationships that could be construed as a potential conflict of interest.

## Publisher’s Note

All claims expressed in this article are solely those of the authors and do not necessarily represent those of their affiliated organizations, or those of the publisher, the editors and the reviewers. Any product that may be evaluated in this article, or claim that may be made by its manufacturer, is not guaranteed or endorsed by the publisher.
